# Granulomatous panuveitis in disseminated sporotrichosis: case report and review of the literature

**DOI:** 10.1186/s12348-023-00330-9

**Published:** 2023-03-09

**Authors:** Wijak Kongwattananon, Tanavadee Rattanaphong

**Affiliations:** Center of Excellence in Retina, Department of Ophthalmology, Faculty of Medicine, Chulalongkorn University and King Chulalongkorn Memorial Hospital, Thai Red Cross Society 1873,, Rama 4 Road, Pathumwan, Bangkok, 10330 Thailand

**Keywords:** Sporotrichosis, *Sporothrix*, Fungal endophthalmitis, Panuveitis

## Abstract

**Purpose:**

To report a case of intraocular sporotrichosis presenting with bilateral granulomatous panuveitis.

**Methods:**

Observational case report and literature review.

**Case presentation:**

A 62-year-old woman with a history of polycythemia vera presented with a non-healing ulcer at the left index finger, generalized erythematous papules, and bilateral granulomatous panuveitis. Cultures of skin and amputated finger identified *Sporothrix schenckii*. The diagnosis of intraocular sporotrichosis secondary to disseminated sporotrichosis was made. Intravenous liposomal Amphotericin B and intravitreal Amphotericin B were used to control systemic and ocular disease resulting in resolution of the skin lesions and intraocular inflammation.

**Conclusions:**

Intraocular sporotrichosis can manifest as bilateral granulomatous panuveitis in the setting of disseminated sporotrichosis. Treatment with intravenous and intravitreal antifungal therapy is useful for controlling intraocular infection.

## Background

Sporotrichosis is a subacute or chronic mycotic infection caused by dimorphic fungi of the genus *Sporothrix*found in soil and plant material. The disease is prevalent worldwide, but more commonly reported in tropical and subtropical regions such as countries in Asia, Oceania, Central and South America and Africa [1]. The transmission generally occurs by traumatic inoculation of the fungus into the skin. Zoonotic transmission through scratching and biting by infected cats has been reported in highly endemic areas such as Brazil [2]. Less frequently, sporotrichosis may be acquired through inhalation of spores of the fungus from the environment. Sporotrichosis primarily involves subcutaneous tissues, along with adjacent lymphatic vessels. Extracutaneous forms including intraocular infection have been reported in the setting of disseminated infection, particularly in immunosuppressed individuals [3–5].

We report a rare case of disseminated sporotrichosis presenting with bilateral granulomatous panuveitis which was successfully treated with intravenous and intravitreal antifungal therapy.

## Case report

A 62-year-old Thai woman was referred to our uveitis clinic for evaluation of blurring of vision in both eyes for a week. She had a 5-year history of polycythemia vera currently treated with hydroxyurea (500 mg/day). Her past ocular history was unremarkable. 1 month before the onset of ocular complaint, she reported trauma to the left index finger by a harrow while gardening which resulted in a non-healing wound. She received multiple debridements along with a course of oral Amoxycillin and clavulanic acid, but it did not improve. Over the past week, she also developed generalized skin lesions involving her face, trunk, and extremities.

**Fig. 1:**
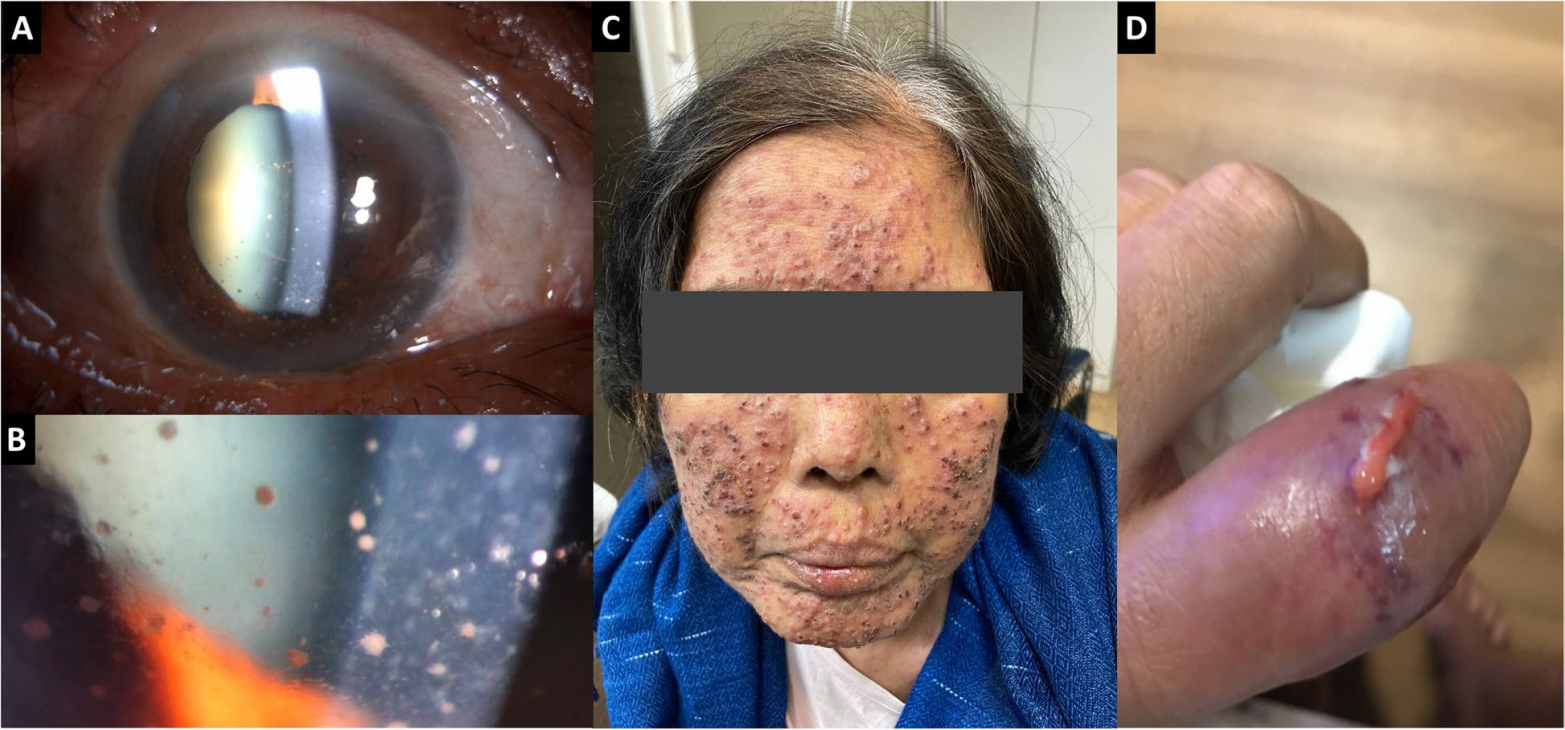
Slit-lamp photograph of the right eye (**A**), higher magnification (**B**) showing diffuse granulomatous keratic precipitates predominantly at the inferior cornea. Clinical photograph of the face (**C**) showing multiple diffuse erythematous papules with vesicles. Clinical photograph of the left index finger (**D**) showing swollen soft tissue and pus discharge

**Fig. 2:**
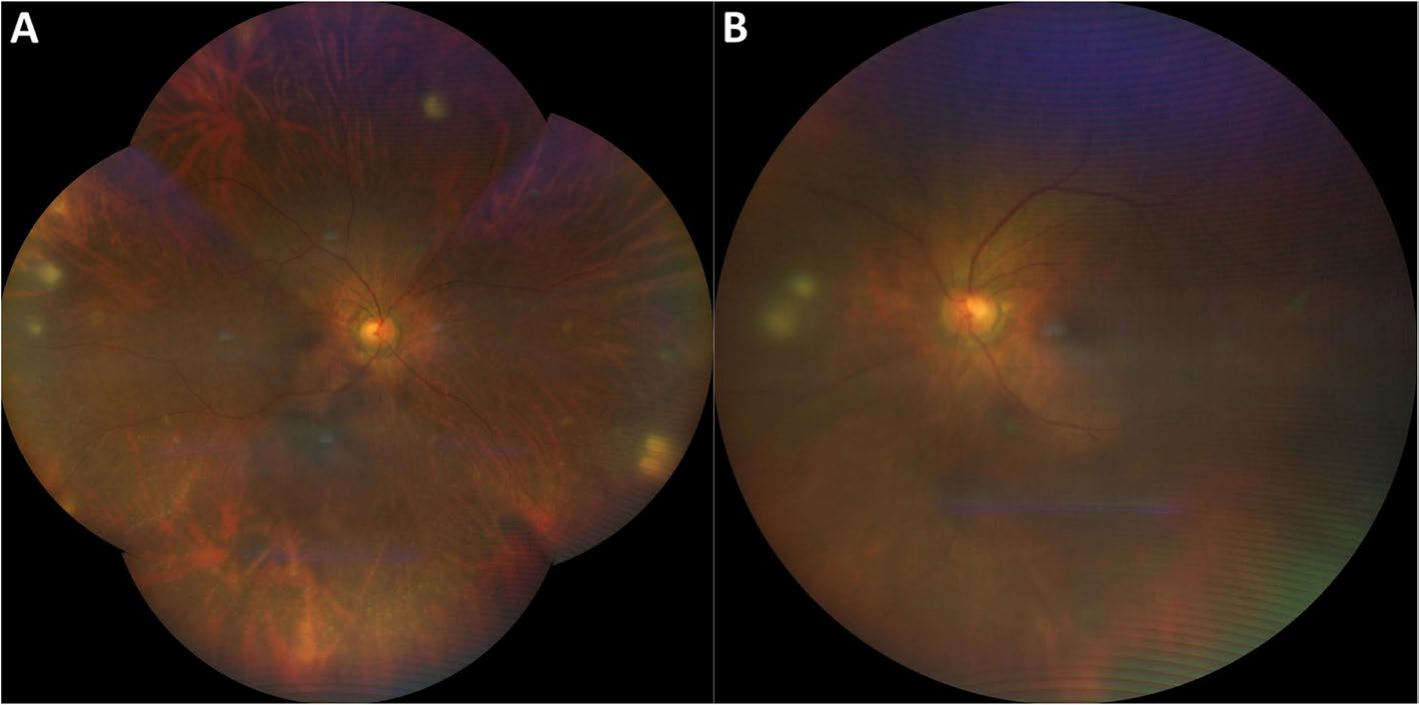
Color fundus photographs of the right (**A**) and left (**B**) eye showing multiple round choroiditis lesions

At the initial ocular examination, her best-corrected visual acuity (BCVA) was 20/125 in the right eye and 20/100 in the left eye. The intraocular pressure was normal in each eye. Slit lamp biomicroscopy of both eyes revealed mild conjunctival injection, granulomatous keratic precipitates (KPs) predominantly involved inferior cornea (Fig. 1A, B), 3 + cells in the anterior chamber, posterior synechiae, and 3 + anterior vitreous cellular reaction. Fundus examination of the right eye showed mild vitritis and multiple diffuse small yellowish chorioretinal lesions at the periphery. (Fig. 2A) The left eye revealed moderate vitritis and chorioretinal lesions similar to the right eye (Fig. 2B).

**Fig. 3:**
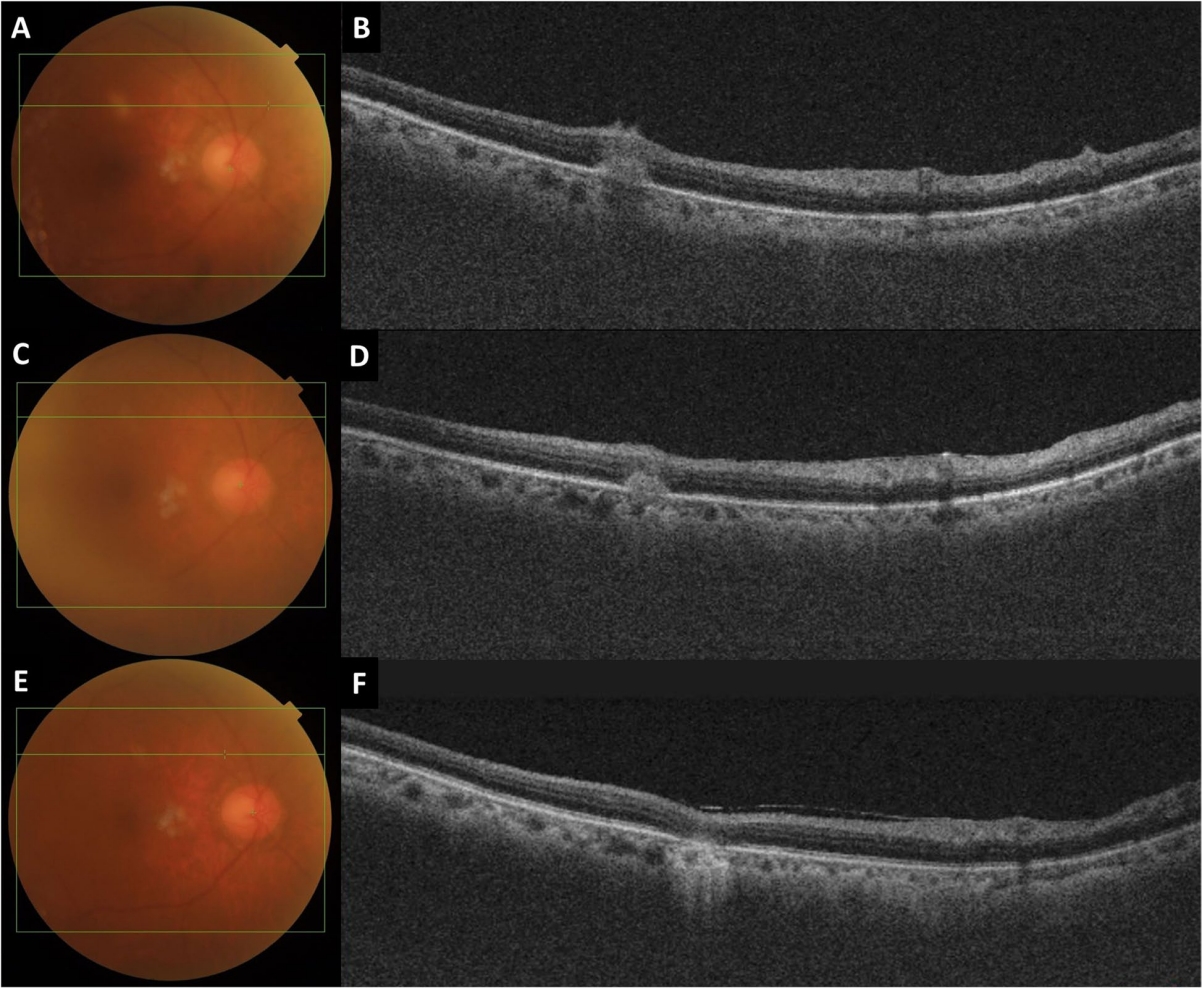
Serial fundus photographs and SS-OCT scans passing through a choroiditis lesion of the right eye showing a dome-shaped hyper-reflective lesion involving outer and inner retina at baseline. **A**, **B** At 2-month follow-up, the lesion became more well-circumscribed and decreased in size (**C**, **D**). At 6-month follow-up, the lesion disappeared, resulting in loss of outer retinal layer

Swept-source optical coherence tomography (SS-OCT) scan passing through a lesion in the right eye demonstrated an ill-defined dome-shaped hyper-reflective lesion overlying the retinal pigment epithelium (RPE) extending to the inner retina (Fig. 3). OCT in the left eye was not obtained due to poor signal from vitritis.

The patient was initially evaluated by the internist. Skin examination was notable for multiple erythematous umbilicated papules with pustules scattering at her face (Fig. 1C), extremities, and trunk. A swollen left index finger with pus discharge on top of the ulcer was also noted (Fig. 1D). Other physical findings were within normal limits. Disseminated candidiasis was suspected at that time. Direct examination with KOH and Tzanck smear of the skin lesion was negative. The patient was admitted and empirically treated with intravenous Amphotericin B (35 mg/day). Complete blood count showed white blood cells of 15,240/mm^3^ with 90% neutrophils and 5% lymphocytes. Her hemoglobin was 9.0 g/dl and platelets were 471,000/mm^3^. Systemic workups to rule out infection causes, including bacterial and fungal hemoculture, anti-HIV, interferon-gamma release assay, and syphilis serology were performed and all of which was negative. The chest X-ray showed no abnormality. Left aqueous sample was sent for PCR fungus and the result was negative.

**Fig. 4:**
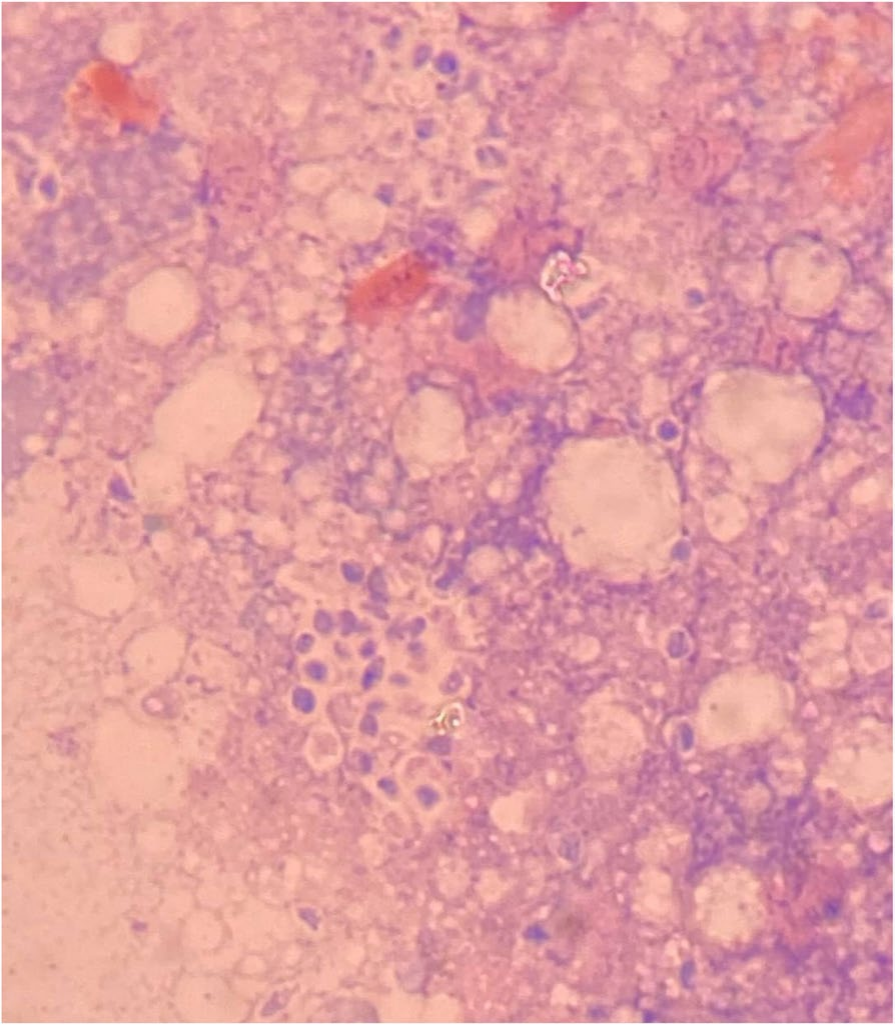
Wright stain of biopsy-tissue of the leg showing intracellular and extracellular yeast

During the admission, a skin biopsy was performed on nodules of the left leg and cheek. Both of them showed numerous yeast cells on Wright stain (Fig. 4) and grew *sporothrix* spp. in fungal cultures using Sabouraud dextrose agar. *Sporothrix schenckii*. was later identified on mold colony by PCR method. The fungal DNA identification was performed by sequencing internal transcribed spacer (ITS) and calmodulin (CAL) gene.

Acute osteomyelitis at her index finger was diagnosed by an orthopedist and was eventually amputated due to the progression despite treatment. The culture of amputated tissue with Sabouraud dextrose agar and PCR also confirmed *Sporothrix schenckii*.

The diagnosis of disseminated sporotrichosis was made. The patient was on intravenous Amphotericin B (35 mg/day) for 14 days and later switched to intravenous liposomal Amphotericin B (150 mg/day) due to acute kidney injury secondary to Amphotericin B toxicity. She completed 8 weeks of treatment with liposomal Amphotericin B and maintained oral itraconazole solution (200 mg/day) treatment 10 months after discharge. A total of five weekly intravitreal Amphotericin B injections (0.05 mg/0.1 ml) were delivered to each eye over 2 months. 1% prednisolone acetate was prescribed for anterior chamber inflammation along with 1% atropine eye drop for breaking posterior synechiae.

Sequential follow-up examinations showed gradual resolution of choroiditis without signs of recurrent intraocular inflammation. Skin lesions also resolved.

At the latest follow-up, BCVA was 20/30 in both eyes. Fundus examination of both eyes revealed no vitreous opacity. Chorioretinal lesions became atrophic. On OCT, the presence of focal disruption of ellipsoid zone and resolution of hyper-reflective lesion was noted (Fig. 3).

## Discussion

Ocular sporotrichosis is uncommon and can occur in healthy individuals or immunosuppressed patients. It can manifest in the form of extraocular or intraocular sporotrichosis. In extraocular form, most reports described the involvement of the conjunctival and ocular adnexa associated with traumatic fungal inoculation. These include granulomatous conjunctivitis, dacryocystitis, and Perinuad’s oculoglandular syndrome [6–8]. In contrast, intraocular form of sporotrichosis has been rarely reported and may result from hematogenous dissemination or direct extension from periocular lesions. Clinically, intraocular sporotrichosis can manifest as endophthalmitis, granulomatous uveitis, retinal granuloma, and multifocal choroiditis [3, 5, 9–15].

In a recent retrospective study of 120 cases with ocular sporotrichosis from Brazil, the majority of patients (75.8%) has isolated ocular manifestation [6]. The most common ocular presentation was granulomatous conjunctivitis (86.7%) mainly affected tarsal conjunctiva followed by eyelid lesions (25%) such as eyelid granuloma, and acute dacryocystitis (7.5%) [6]. Ocular with cutaneous involvement was observed in 24.2% of patients [6]. Among these, fixed cutaneous form was the most prevalent (48.3%) with face (41.4%) and hands (37.9%) being commonly affected sites [6]. In their study, it is also suggested that the fungus is potentially transmitted from skin lesions to the eye either by proximity or rubbing the eye [6].

Previously published intraocular sporotrichosis case reports are summarized in Table 1. The definitive diagnosis of ocular sporotrichosis is obtained by the identification of the fungus from ocular fluid or tissue via cultures, histopathology, or molecular analysis.

**Table 1:** Summary of published cases reports of intraocular sporotrichosis

Author, year	Age/sex	Country	Risk factors	Ocular features	Extraocular Manifestations	Diagnostic Method	Treatment	Outcome
Cassady et al.,1971 [11]	50/M	US	None	Non granulomatous uveitis	None	-Histopathology enucleated eye	Topical, subconjuctiva, systemic steroids	Enucleated
Font et al., 1976 [3]	42/M	US	Alcoholism and DM	Necrotizing retinochoroiditis	None	-Histopathology enucleated eye -Immunofluorescence Electron microscopic studies	Steroids, pyremethamine	Enucleated
Kurosawa et al., 1986 [15]	30 M	US	HIV	Endophthalmitis	Multiple indurated, erythematous papules	-Cultures of aqueous -Histopathology enucleated eye	IV and IVT AMB, kanamycin sulfate, and amikacin sulfate	Enucleated
Cartwright et al. 1993 [10]	24/M	US	Previous injection of periocular steroid	Endophthalmitis	None	-Culture of wound and vitreous	IVT AMB, subconjunctiva AMB	Phthisis
D. Vieira-Dias et al., 1997 [14]	12/F	Brazil	None	Granulomatous panuveitis	Erythematous hardened nodules with lymphatic distribution at the arm	-Cultures of aqueous and skin	Topical AMB, fluconazole, potassium iodided	Phthisis
Curi et al., 2003 [12]	18/ M	Brazil	None	Retinal granuloma	Generalized ulcerated skin lesions	-Cultures of the skin lesion and lymph nodes	IV AMB	Resolved
Silva-Vergara et al.. 2012 [5]	32/ M	Brazil	HIV	Bilateral endophthalmitis	Endocarditis	-Cultures of subcutaneous nodule and mitral valve fragments	IV AMB, oral ITR	Blindness
Biancardi et al., 2017 [9]	35/M	Brazil	HIV	Multifocal choroiditis	Cutaneous, osteoarticular, oral, and mucosa	-Cultures of blood, bone a marrow aspirate, lymph nodes, BAL fluid	IV AMB	Cured
	25/M	Brazil	HIV	Multifocal choroiditis	Cutaneous, osteoarticular, pulmonary, bone marrow, and lymph node	-Cultures of blood, larynx, pharynx, and oral mucosa	IV AMB	Cured
	43/M	Brazil	HIV	Multifocal choroiditis	Cutaneous and osteoarticular	-Cultures of blood, nasal mucosa, and sputum	IV AMB	Cured
Mohd Rasidin et al., 2022 [13]	43/M	Malaysia	None	Granulomatous anterior uveitis	Multiple subcutaneous nodular lesions at the forearm	-Clinical diagnosis	Oral ITR	Resolved

*AMB* Amphotericin B, *BAL* Bronchoalveolar lavage, *IV* Intravenous, *IVT* Intravitreal, *ITR* Itraconazole

Disseminated sporotrichosis usually occurs in an individual with impaired cellular immunity such as patients living with human immunodeficiency virus (PLHIV), alcoholism, or the use of immunosuppressive medication [16, 17]. The cutaneous system is the most commonly affected in disseminated sporotrichosis and typically presents as generalized ulcerated nodular lesions and verrucous plaques [18]. Extracutaneous involvement reported includes lungs, bones, joints, spleen, intestine, central nervous system, and intraocular inflammation [17].

The primary risk factor of *sporothrix*infection found in our case is a skin injury caused by harrow 1 month before the beginning of the cutaneous and ocular disease. The use of long-term hydroxyurea is an important risk factor for disseminated infection. Hydroxyurea is an anti-cancer drug that works by inhibiting DNA replication. As a result, T cells may be impaired to expand and respond to sporotrichosis infection [19]. In the present case, culture-proven cutaneous disseminated sporotrichosis allowed us to diagnose the patient with intraocular sporotrichosis despite the negative result of aqueous PCR for fungal as the yield may be low.

This case is one of few reports of non-HIV patients with concurrent intraocular and cutaneous sporotrichosis. Curi et al. [12] described an 18-year-old Brazilian man presenting with retinal granuloma and cutaneous disseminated sporotrichosis. In a case report by D. Vieira-Dias et al. [14], a 12-year-old Brazilian girl had clinical endophthalmitis following painful erythematous nodular lesions on her arm. *Sporothrix* was positive in the culture of aqueous*.* Recently, Raja ther R et al. [13] reported a 43-year-old man with granulomatous anterior uveitis who had multiple subcutaneous nodular lesions suggestive of sporotrichosis. These previous cases are different from ours as their patients were without comorbidities and no causes of immunosuppression were identified. Also, the latter two cases had cutaneous-lymphatic sporotrichosis form, limiting to the arm or forearm.

OCT was helpful in making diagnosis and monitoring treatment response in the present case. At the first evaluation, the patient’s OCT scan passing through the area of choroiditis revealed dome-shaped elevation of the outer retina and pigment epithelium with overlying hyper-reflectivity involving the inner retinal layer. This characteristic was quite similar to the lesions seen in other fungal choroiditis such as candida [20]. On follow-up OCT, the lesions were found to be well-circumscribed in response to antifungal therapy.

According to the guideline for the management of sporotrichosis by the Infectious Diseases Society of America (IDSA) [4], the recommended treatment for disseminated sporotrichosis involves intravenous Amphotericin B preferable in the lipid formulation which is given at a dose of 3–5 mg/kg daily as loading therapy followed by a course of oral itraconazole at a dose of 200 mg twice a day for at least 12 months. For patients with comorbidities or with immunosuppression, long-term or life-long systemic antifungal treatment may be required [4]. In cases of intraocular involvement such as endophthalmitis, intravitreal Amphotericin B can be used as an adjunctive treatment [10, 15]. The visual prognosis of intraocular sporotrichosis largely remains poor in most previously published cases, mainly due to delay in diagnosis and late starting of antifungal. The affected eyes usually deteriorate resulting in blindness and phthisis [3, 5, 10, 11, 15]. Our immunocompromised patient had significant vitritis and multiple choroiditis bilaterally. We recommend treatment with the combination of intravitreal Amphotericin B and systemic antifungal therapy to ensure adequate drug levels in the vitreous are maintained. Regarding treatment of osteomyelitis in disseminated sporotrichosis, it should be noted that amputation of the finger or bone involved is not standard management of this condition. Alternatively, it can be initially managed with saturated solution of potassium iodine (SSKI), systemic antifungal treatment, and debridement [21]. A consultation with an orthopedic surgeon should also be considered.

## Conclusions

This case illustrates that intraocular sporotrichosis can manifest as bilateral granulomatous panuveitis as a result of disseminated sporotrichosis. It should be suspected in an immunosuppressed individual who has a recent history of skin trauma by organic matter and the presence of characteristics of disseminated skin lesions. For all patients diagnosed with disseminated sporotrichosis, we recommend performing a thorough ophthalmic examination to early detect intraocular sporotrichosis and promptly treat it as late treatment might result in a poor visual outcome. The diagnosis can be challenging as it often requires microbiological evidence from extraocular tissues. A combination of systemic and intravitreal Amphotericin B can be used to treat intraocular sporotrichosis in the setting of disseminated infection.

## Data Availability

Not applicable.
